# Crystal structures of 5-amino-*N*-phenyl-3*H*-1,2,4-di­thia­zol-3-iminium chloride and 5-amino-*N*-(4-chloro­phen­yl)-3*H*-1,2,4-di­thia­zol-3-iminium chloride monohydrate

**DOI:** 10.1107/S2056989015016655

**Published:** 2015-09-12

**Authors:** Chien Ing Yeo, Yee Seng Tan, Edward R. T. Tiekink

**Affiliations:** a Department of Chemistry, University of Malaya, 50603 Kuala Lumpur, Malaysia; b Centre for Chemical Crystallography, Faculty of Science and Technology, Sunway University, 47500 Bandar Sunway, Selangor Darul Ehsan, Malaysia

**Keywords:** crystal structure, hydrogen bonding, di­thia­zole ring, salt

## Abstract

The crystal and mol­ecular structures of the title salt, C_8_H_8_N_3_S_2_^+^·Cl^−^, (I), and salt hydrate, C_8_H_7_ClN_3_S_2_^+^·Cl^−^·H_2_O, (II), are described. The heterocyclic ring in (I) is statistically planar and forms a dihedral angle of 9.05 (12)° with the pendant phenyl ring. The comparable angle in (II) is 15.60 (12)°, indicating a greater twist in this cation. An evaluation of the bond lengths in the H_2_N—C—N—C—N sequence of each cation indicates significant delocalization of π-electron density over these atoms. The common feature of the crystal packing in (I) and (II) is the formation of charge-assisted amino-N—H⋯Cl^−^ hydrogen bonds, leading to helical chains in (I) and zigzag chains in (II). In (I), these are linked by chains mediated by charge-assisted iminium-N^+^—H⋯Cl^−^ hydrogen bonds into a three-dimensional architecture. In (II), the chains are linked into a layer by charge-assisted water-O—H⋯Cl^−^ and water-O—H⋯O(water) hydrogen bonds with charge-assisted iminium-N^+^—H⋯O(water) hydrogen bonds providing the connections between the layers to generate the three-dimensional packing. In (II), the chloride anion and water mol­ecules are resolved into two proximate sites with the major component being present with a site occupancy factor of 0.9327 (18).

## Abstract 2

The five-membered 1,2,4-di­thia­zole rings in the cations of (I) and (II) are almost planar, but in each case the overall cation is twisted with dihedral angles between the planes of the heterocycle and the pendant aryl ring of 9.05 (12) and 15.60 (12)° in (I) and (II), respectively. In both compounds, the bond lengths in the H_2_N—C—N—C—N backbones imply considerable delocalization of π-electron density.

## Chemical context   

The title salts were isolated as a part of a research programme into the crystal engineering aspects and biological potential of phosphanegold(I) carbonimido­thio­ates, *i.e*. mol­ecules of the general formula *R*_3_PAu[SC(O*R*′)=N*R*′′]; *R*, *R*′, *R*′′ = aryl and/or alkyl. While earlier work focussed on supra­molecular aggregation patterns (Kuan *et al.*, 2008 ▸) and solid-state luminescence (Ho *et al.*, 2006 ▸), more recent endeavours have focussed upon biological studies. For example, the Ph_3_PAu[SC(O–alk­yl)=N(*p*-tol­yl)] compounds prove to be very potent against Gram-positive bacteria (Yeo, Sim *et al.*, 2013 ▸). In addition, Ph_3_PAu[SC(O–alk­yl)=N(ar­yl)] com­pounds exhibit significant cytotoxicity and kill cancer cells by initiating a variety of apoptotic pathways (Yeo, Ooi *et al.*, 2013 ▸; Ooi, Yeo *et al.*, 2015 ▸). A focus of recent synthetic efforts has been to increase the functionality of the thio­carbamide mol­ecules in order to produce gold complexes of higher nuclear­ity. During this work bipodal {1,4-[MeOC(=S)N(H)]_2_C_6_H_4_} was synthesized along with its binuclear phosphanegold(I) complexes (Yeo *et al.*, 2015 ▸). As an expansion of these studies, the 1:2 reactions of thio­urea with aryl­iso­thio­cyanates were undertaken which, rather than yielding bipodal mol­ecules, gave the 1:1 cyclization products, isolated as salts. These and related compounds have been described in the patent literature as having a range of biological properties, *e.g*. as bactericides, fungicides and plant-growth inhibitors (Röthling *et al.*, 1989 ▸). Herein, the crystal and mol­ecular structures of two examples of these products, *i.e*. the salt, [C_8_H_8_N_3_S_2_]Cl (I), and the salt hydrate [C_8_H_7_ClN_3_S_2_]Cl·H_2_O (II), are described.
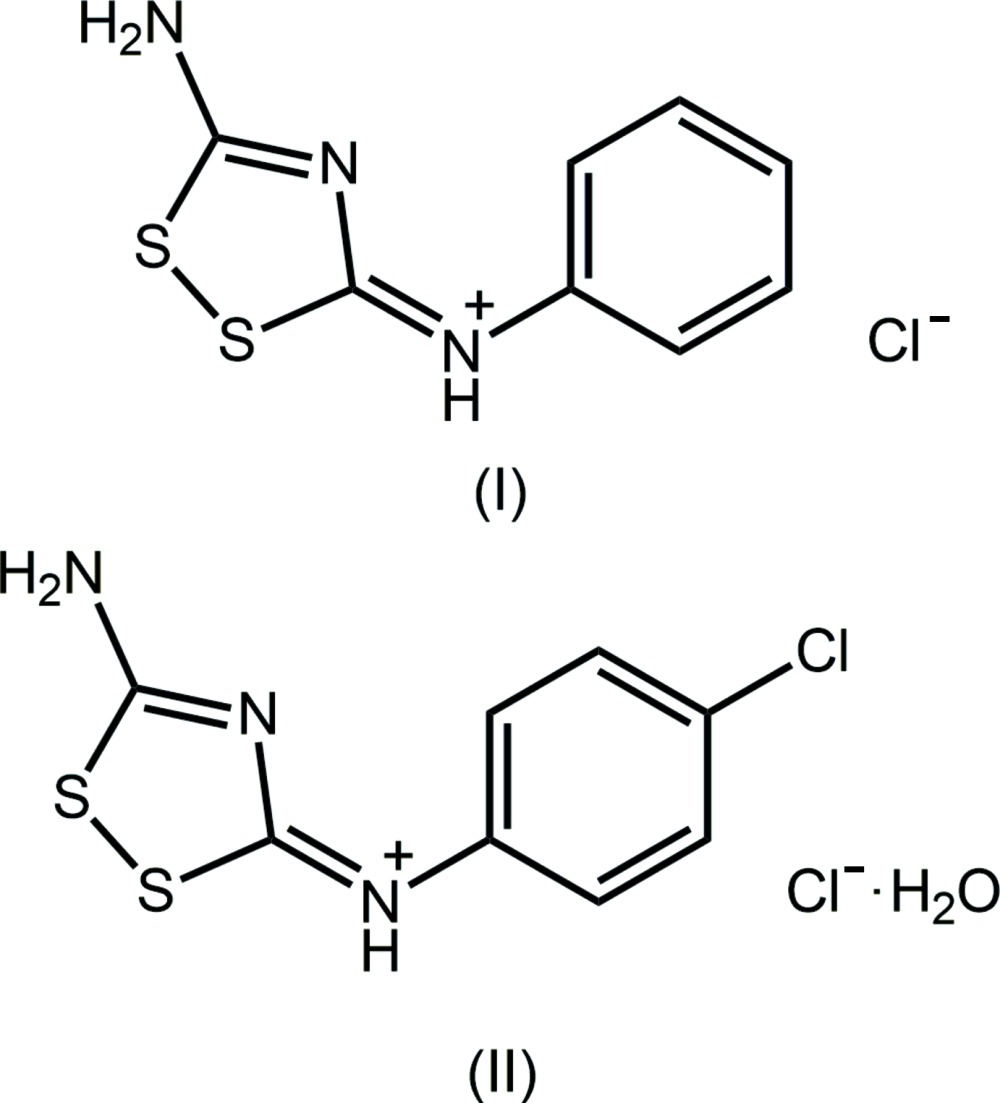


## Structural commentary   

**Figure 1:**
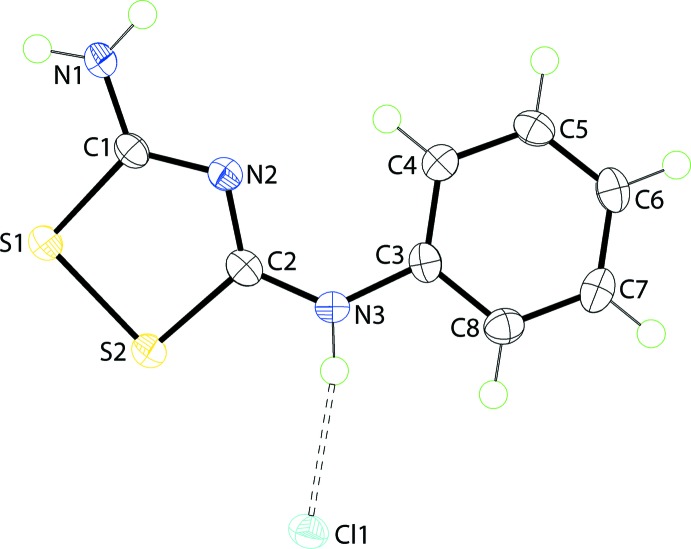
The asymmetric unit for (I), showing the atom-labelling scheme and displacement ellipsoids at the 70% probability level. The dashed lines indicate hydrogen bonds.

The asymmetric unit of (I), comprising a cation and chloride anion, is shown in Fig. 1 ▸. The five-membered 1,2,4-di­thia­zole ring of the cation in (I) is strictly planar with the maximum deviation being less than ±0.003 (2) Å. However, the entire cation is not planar with the dihedral angle between the rings being 9.05 (12)°. Selected geometric parameters are collected in Table 1 ▸. While the S—S and S—C bond lengths correspond to single bonds, an evaluation of the C—N bonds, inter­nal and external to the ring, suggest a high level of delocalization of π-electron density across these atoms. The angles subtended at the S atoms are nearly right-angles. The trigonal angles around the C1 atom are all approximately 120° but there is a range of 10° for the angles about the C2 atom, with the widest angle being N2—C2—N3, consistent with double-bond character in the C—N bonds. The widest angle in the mol­ecule is that subtended at the N3 atom, an observation that correlates with the C2=N3 double bond and the presence of the small H atom on the N3 atom.

**Figure 2:**
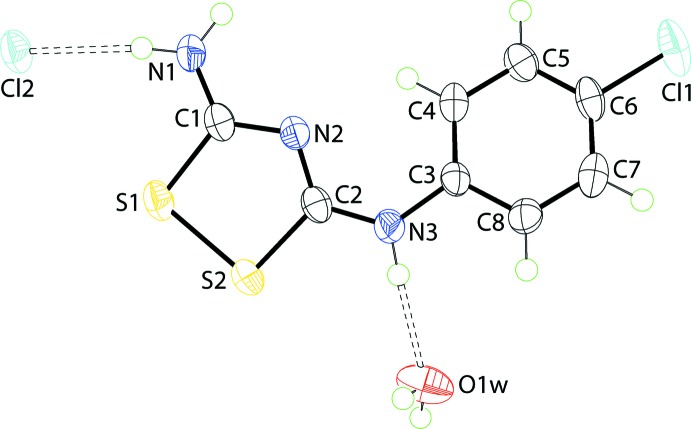
The asymmetric unit for (II), showing the atom-labelling scheme and displacement ellipsoids at the 70% probability level. The dashed lines indicate hydrogen bonds.

The asymmetric unit of (II), comprising a cation, a chloride anion and a water mol­ecule of crystallization, is illustrated in Fig. 2 ▸. As for (I), the cation is almost planar with the maximum deviation being 0.010 (2) Å for the N2 atom; the r.m.s. deviation for the fitted atoms is 0.010 Å. A greater overall twist in the mol­ecule is evident, as seen in the dihedral angle of 15.60 (12)° formed between the rings. In terms of bond lengths, Table 1 ▸, the discussion above for (I), holds true for (II). Similarly, for the bond angles except that the range of angles about the C2 atom is narrower at 6°.

**Figure 3:**
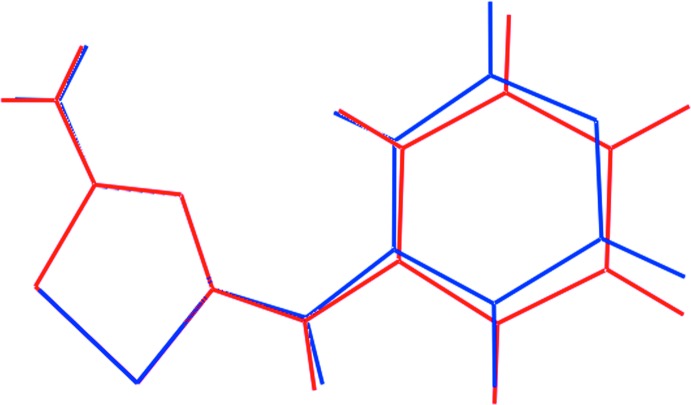
Overlay diagram of the cations in (I), red image, and (II), blue image. The cations have been overlapped so that the five-membered rings are coincident.

Fig. 3 ▸ presents an overlay diagram of the cations in each of (I) and (II) which highlights the similarity in their mol­ecular structures.

## Supra­molecular features   

Geometric parameters characterizing the inter­molecular inter­actions operating in the crystal structures of (I) and (II) are collected in Tables 2 ▸ and 3 ▸, respectively.

**Figure 4:**
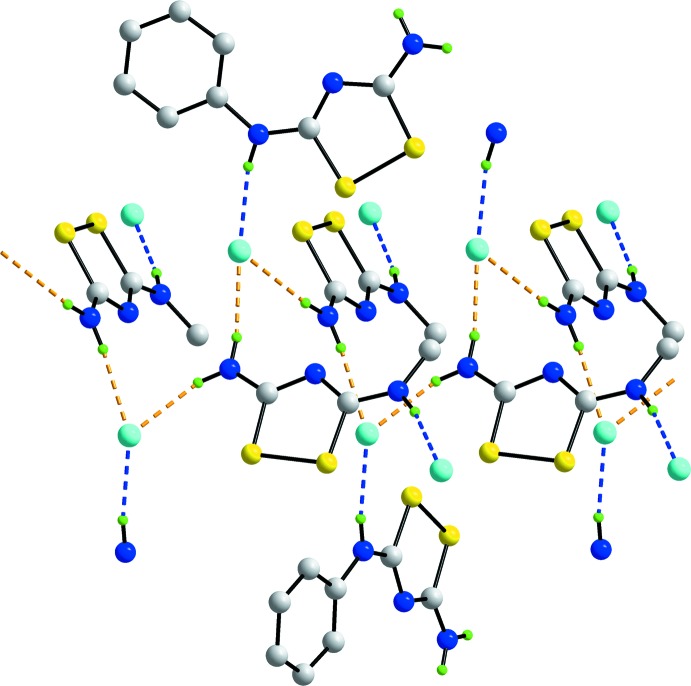
Detail of the hydrogen bonding operating in the crystal structure of (I). The charge-assisted amino-N—H⋯Cl^−^ hydrogen bonds are shown as orange dashed lines and lead to helical chains along [100]. The charge-assisted imino-N^+^—H⋯Cl^−^ hydrogen bonds are shown as blue dashed lines and lead to chains along [011]. For reasons of clarity, H atoms not involved in hydrogen bonding have been omitted and only one of the chains along [011] is shown.

**Figure 5:**
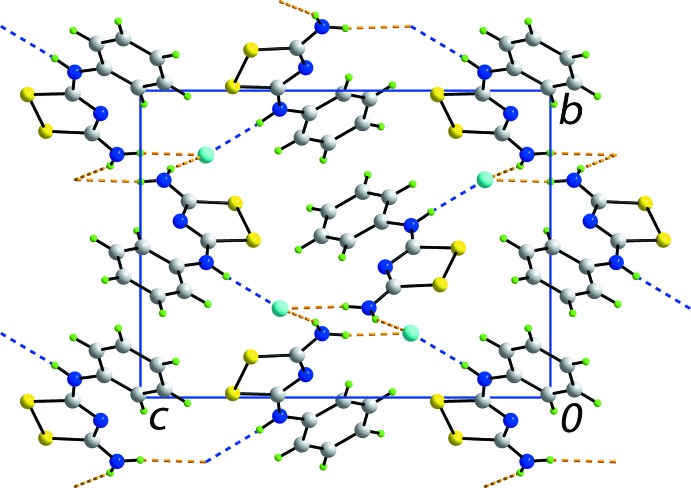
Unit-cell contents for (I) shown in projection down the *a* axis. The charge-assisted amino-N—H⋯Cl^−^ and imino-N^+^—H⋯Cl^−^ hydrogen bonds are shown as orange and blue dashed lines, respectively.

The presence of charge-assisted N—H⋯Cl^−^ and N^+^—H⋯Cl^−^ hydrogen bonds are crucial in establishing the three-dimensional architecture in the crystal structure of (I). The structure is conveniently described as comprising columns of cations aligned along the *a* axis connected through hydrogen bonds to rows of chloride ions, also aligned along the *a* axis. As illustrated in Fig. 4 ▸, charge-assisted amino-N—H⋯Cl^−^ hydrogen bonds lead to helical chains along [100], being generated by 2_1_ screw symmetry. The chains are linked to neighbouring chains by charge-assisted iminium-N^+^—H⋯Cl^−^ hydrogen bonds, that in themselves lead to chains aligned along [011]. In this way, a three-dimensional architecture is constructed as shown in projection in Fig. 5 ▸.

**Figure 6:**
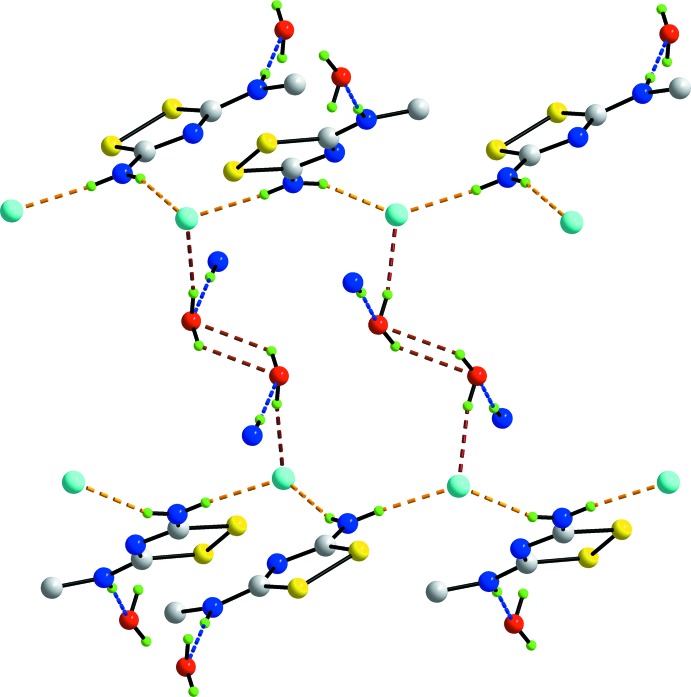
Detail of the hydrogen bonding operating in the crystal structure of (II). The charge-assisted amino-N—H⋯Cl^−^ hydrogen bonds are shown as orange dashed lines and lead to zigzag chains along [001]. The charge-assisted imino-N^+^—H⋯O(water) hydrogen bonds are shown as blue dashed lines and both water-O—H⋯Cl^−^ and water-O—H⋯O(water) hydrogen bonds are shown as brown dashed lines. For reasons of clarity, H atoms not involved in hydrogen bonding have been omitted.

**Figure 7:**
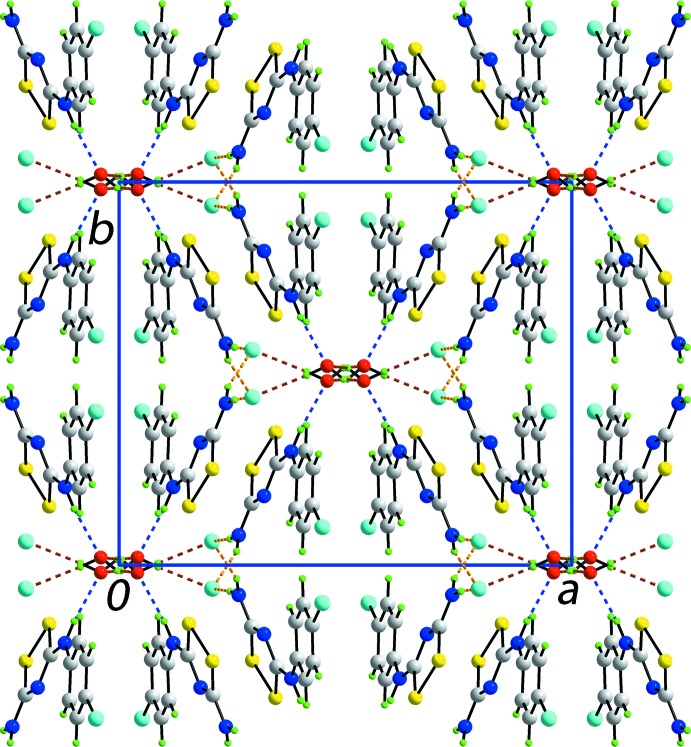
Unit cell contents for (II) shown in projection down the *c* axis. The charge-assisted amino-N—H⋯Cl^−^ (orange), imino-N^+^—H⋯Cl^−^ (blue), water-O—H⋯Cl^−^ (brown) and water-O—H⋯O(water) (brown) hydrogen bonds are shown as dashed lines.

A more complicated pattern of hydrogen bonding occurs in the crystal structure of (II). The amino-H atoms form charge-assisted N—H⋯Cl^−^ hydrogen bonds while the iminium-H atom forms a charge-assisted N^+^—H⋯O hydrogen bond to the water mol­ecule of crystallization. The water mol­ecule also forms two donor inter­actions, one to another water mol­ecule and the second, charge-assisted, to the chloride anion. Hence, all donor atoms participate in the hydrogen-bonding scheme and each of the chloride and water species forms three hydrogen bonds. A diagram showing the detail of the hydrogen bonding is shown in Fig. 6 ▸. The amino-N—H⋯Cl^−^ bridges clearly persist, as for (I), but lead to zigzag chains (glide symmetry) along the *c* axis. As pairs of water mol­ecules are linked *via* water-O—H⋯O(water) hydrogen bonds across a centre of inversion and each forms a charge-assisted water-O—H⋯Cl^−^ hydrogen bond, the water mol­ecules form links between the zigzag chains resulting in supra­molecular layers. Finally, the water mol­ecules accept charge-assisted imino-N^+^—H⋯O(water) hydrogen bonds, providing links between the layers so that a three-dimensional architecture ensues. As seen from Fig. 7 ▸, globally, the structure may be described as comprising layers of cations parallel to [001] that define rectangular channels parallel to [001] incorporating the anions and inter­nalized water mol­ecules. Not shown in Fig. 5 ▸, are indications of close Cl1⋯Cl1^i^ contacts of 3.3510 (10) Å which occur within layers rather than between layers; symmetry operation (ii): 1 − *x*, *y*, −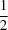 − *z*.

## Database survey   

A search of the Cambridge Structural Database (Groom & Allen, 2014 ▸), revealed there are no direct analogues of (I) and (II) in the crystallographic literature. The structure of a closely related neutral species, *i.e*. 5-(di­methyl­amino)-3-(phenyl­imino)-1,2,4-di­thia­zole, characterized in its 1:1 co-crystal with 2-(di­methyl­carboxamido-imino)­benzo­thia­zole, (III) in the scheme below, has been reported (Flippen, 1977 ▸), along with several benzoyl derivatives, as exemplified by 3-(4-methyl-benzoyl­imino)-5-phenyl­amino-3*H*-1,2,4-di­thia­zole (IV) (Kleist *et al.*, 1994 ▸). An evaluation of the bond lengths in the N—C—N—C—N sequences in these mol­ecules suggests a greater contribution of the canonical structure with formal C=N bonds, *i.e*. N—C=N—C=N. This difference is traced to the influence of the formal charge on the iminium-N atom.
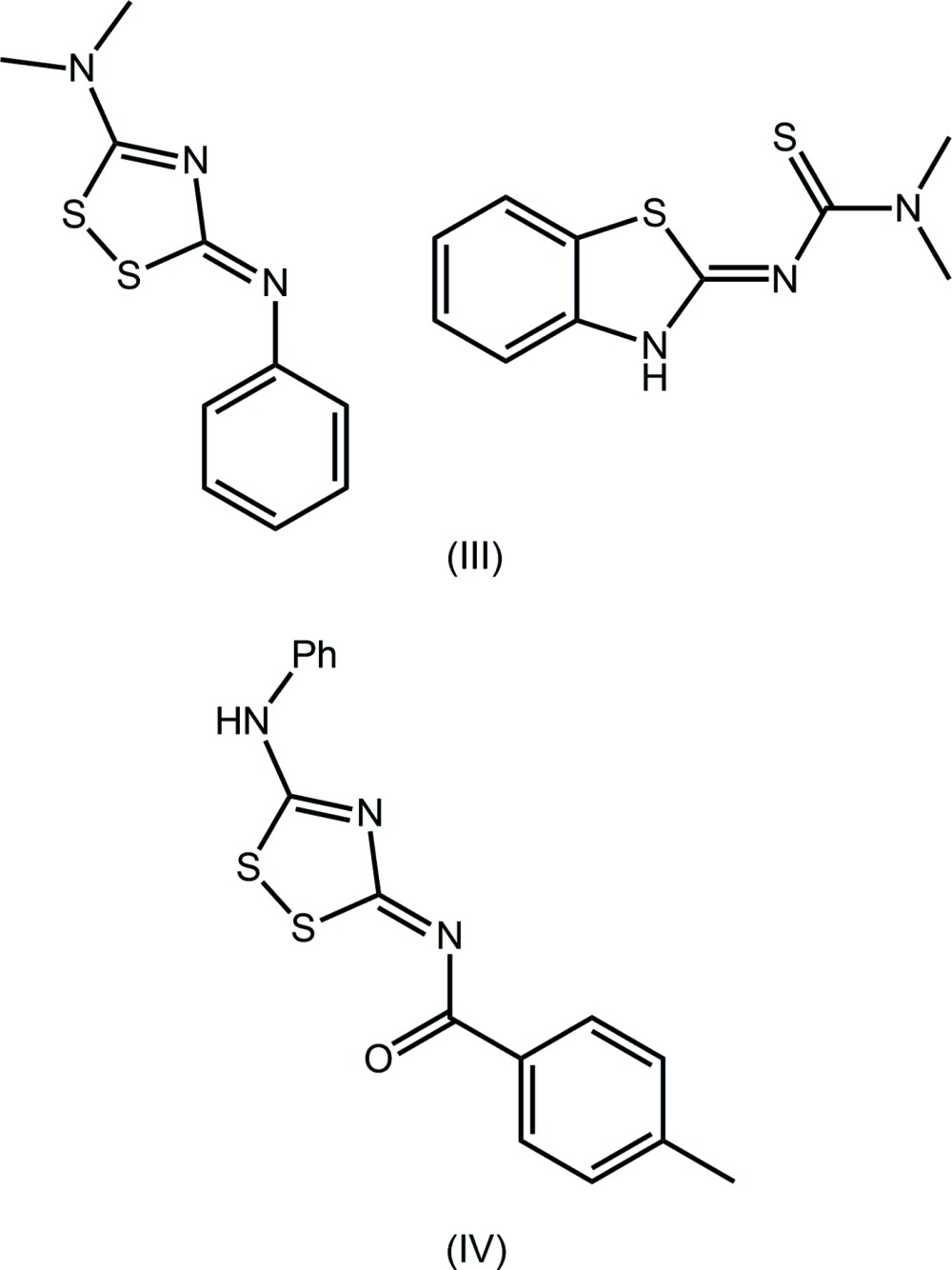


## Synthesis and crystallization   

**Synthesis of (I).** To thio­urea (Merck, 5 mmol, 0.38 g) in aceto­nitrile (20 ml) was added 50% *w*/*v* NaOH (10 mmol, 0.40 ml) and phenyl iso­thio­cyanate (Merck, 10 mmol, 1.2 ml). The resulting mixture was stirred for 4 h at 323 K. 5 *M* HCl (20 mmol, 4.1 ml) was added and the mixture was stirred for another 1 h. The final product was extracted using chloro­form (200 ml). The powder that formed after 2 weeks was re-dissolved in dichoro­methane/aceto­nitrile (1:1 *v*/*v*, 200 ml), yielding yellow prisms after 3 weeks. Yield: 0.627 g (51%). M.p. 492–493 K. ^1^H NMR (400 MHz, DMSO-*d*_6_, 298 K): 13.37 (*s*, *br*, 1H, NH), 10.73 (*s*, 1H, NH_2_), 10.66 (*s*, *br*, 1H, NH_2_), 7.74 (*d*, 2H, *o*-Ph-H, *J* = 7.96 Hz), 7.45 (*dd*, 2H, *m*-Ph-H, *J* = 7.82 Hz, *J* = 7.82 Hz), 7.27 (*t*, 1H, *p*-Ph-H, *J* = 7.34 Hz). ^13^C NMR (400 MHz, DMSO-*d*_6_, 298 K): 182.9 [SC(=N)N], 176.1 [C(NH_2_)], 138.5 (C_*ipso*_), 129.7 (C_*meta*_), 126.5 (C_*para*_) 121.4 (C_*ortho*_). IR (cm^−1^): 3414 (*m*) (N—H), ν 3007 (*m*) (C—H), ν 1248 (*s*) (C—N).

**Synthesis of (II).** The *p*-chloro derivative (II) was prepared as described above but using 4-chloro­phenyl iso­thio­cyanate (Sigma–Aldrich) as the unique reagent. Yellow prismatic crystals were isolated after 4 weeks. Yield: 0.581 g (39%). M.p. 484–485 K. ^1^H NMR (400 MHz, DMSO-*d*_6_, 298 K): 13.51 (*s*, *br*, 1H, NH), 10.72 (*s*, 1H, NH_2_), 10.41 (*s*, *br*, 1H, NH_2_), 7.77 (*d*, 2H, *m*-Ph-H, *J* = 8.60 Hz), 7.52 (*d*, 2H, *o*-Ph-H, *J* = 8.52 Hz), 3.48 (*br*, 2H, H_2_O). ^13^C NMR (400 MHz, DMSO-*d*_6_, 298 K): 183.0 [SC(=N)N], 176.1 [CNH_2_], 137.4 (C_*ipso*_), 130.3 (C_*para*_), 129.6 (C_*meta*_), 122.9 (C_*ortho*_). IR (cm^−1^): ν 2965 (*br*) (O—H), ν 1250 (s) (C—N).

## Refinement   

Crystal data, data collection and structure refinement details are summarized in Table 4 ▸. For (I) and (II), carbon-bound H atoms were placed in calculated positions (C—H = 0.95 Å) and were included in the refinement in the riding-model approximation, with *U*_iso_(H) set to 1.2*U*_eq_(C). The N-bound H-atoms were located in a difference Fourier map but were refined with a distance restraint of N—H = 0.88±0.01 Å, and with *U*_iso_(H) set to 1.2*U*_eq_(N). For (I), owing to poor agreement, one reflection, *i.e*. (020), was omitted from the final cycles of refinement. For (II), disorder was noted in the structure, involving the Cl2 anion and water mol­ecule of crystallization so that two proximate positions were resolved for the heteroatoms. The major component refined to a site occupancy factor of 0.9327 (18). The anisotropic displacement parameters for the pair of Cl2 anions and for the water-O atoms were constrained to be equal. Only the water-bound H atoms for the major component were resolved and these were assigned full weight with O—H 0.84±0.01 Å, and with *U*_iso_(H) = 1.5*U*_eq_(O).

## Supplementary Material

Crystal structure: contains datablock(s) I, II, global. DOI: 10.1107/S2056989015016655/hb7500sup1.cif

Structure factors: contains datablock(s) I. DOI: 10.1107/S2056989015016655/hb7500Isup2.hkl

Structure factors: contains datablock(s) II. DOI: 10.1107/S2056989015016655/hb7500IIsup3.hkl

Supporting information file. DOI: 10.1107/S2056989015016655/hb7500Isup4.cml

Supporting information file. DOI: 10.1107/S2056989015016655/hb7500IIsup5.cml

CCDC references: 1422604, 1422603

Additional supporting information:  crystallographic information; 3D view; checkCIF report

## Figures and Tables

**Table 1:** Geometric data (Å, °) for (I) and (II)

Parameter	(I)	(II)
S1—S2	2.0669 (10)	2.0657 (12)
S1—C1	1.769 (3)	1.749 (3)
S2—C2	1.772 (3)	1.763 (3)
N1—C1	1.309 (3)	1.305 (4)
N2—C1	1.328 (3)	1.337 (4)
N2—C2	1.317 (3)	1.312 (4)
N3—C2	1.328 (3)	1.332 (4)
N3—C3	1.418 (3)	1.424 (4)
		
C1—S1—S2	92.63 (9)	92.68 (11)
C2—S2—S1	92.72 (10)	92.85 (11)
C2—N2—C1	115.1 (2)	115.1 (2)
C2—N3—C3	130.4 (2)	128.0 (3)
N1—C1—N2	122.5 (2)	120.8 (3)
N1—C1—S1	117.8 (2)	119.5 (2)
N2—C1—S1	119.7 (2)	119.8 (2)
N2—C2—N3	125.2 (2)	123.4 (3)
N2—C2—S2	119.8 (2)	119.6 (2)
N3—C2—S2	115.1 (2)	117.0 (2)

**Table 2:** Hydrogen-bond geometry (Å, °) for (I)

*D*—H⋯*A*	*D*—H	H⋯*A*	*D*⋯*A*	*D*—H⋯*A*
N1—H1*N*⋯Cl1^i^	0.87 (2)	2.36 (2)	3.215 (2)	170 (3)
N1—H2*N*⋯Cl1^ii^	0.88 (2)	2.29 (2)	3.131 (3)	159 (3)
N3—H3*N*⋯Cl1	0.88 (2)	2.22 (2)	3.084 (2)	169 (2)

Symmetry codes: (i) 
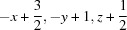
; (ii) 
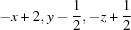
.

**Table 3:** Hydrogen-bond geometry (Å, °) for (II)

*D*—H⋯*A*	*D*—H	H⋯*A*	*D*⋯*A*	*D*—H⋯*A*
N1—H1*N*⋯Cl2^i^	0.88 (3)	2.30 (3)	3.144 (3)	161 (3)
N1—H2*N*⋯Cl2	0.88 (2)	2.22 (1)	3.089 (2)	172 (4)
N3—H3*N*⋯O1*W*	0.88 (2)	2.06 (2)	2.927 (4)	174 (3)
O1*W*—H2*O*⋯O1*W* ^ii^	0.84 (3)	2.29 (4)	2.884 (4)	128 (3)
O1*W*—H1*O*⋯Cl2^iii^	0.85 (3)	2.16 (3)	3.005 (3)	170 (3)

Symmetry codes: (i) 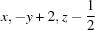; (ii) 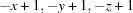; (iii) 
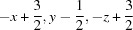
.

**Table 4:** Experimental details

	(I)	(II)
Crystal data		
Chemical formula	C_8_H_8_N_3_S_2_^+^·Cl^−^	C_8_H_7_ClN_3_S_2_^+^·Cl^−^·H_2_O
*M*_r_	245.74	298.20
Crystal system, space group	Orthorhombic, *P*2_1_2_1_2_1_	Monoclinic, *C*2/*c*
Temperature (K)	100	100
*a*, *b*, *c* (Å)	6.5702 (4), 10.8637 (7), 14.4964 (10)	17.0581 (7), 14.1660 (7), 10.3215 (4)
α, β, γ (°)	90, 90, 90	90, 101.084 (4), 90
*V* (Å^3^)	1034.70 (12)	2447.61 (19)
*Z*	4	8
Radiation type	Mo *K*α	Mo *K*α
μ (mm^−1^)	0.73	0.85
Crystal size (mm)	0.15 × 0.02 × 0.02	0.20 × 0.10 × 0.05

Data collection		
Diffractometer	Bruker SMART APEX CCD diffractometer	Agilent SuperNova Dual diffractometer with an Atlas detector
Absorption correction	Multi-scan (*SADABS*; Sheldrick, 1996 ▸)	Multi-scan (*CrysAlis PRO*; Agilent, 2012 ▸)
*T*_min_, *T*_max_	0.898, 1.000	0.748, 1.000
No. of measured, independent and observed [*I* > 2σ(*I*)] reflections	9875, 2378, 2185	19709, 2821, 2142
*R*_int_	0.044	0.064
(sin θ/λ)_max_ (Å^−1^)	0.650	0.650

Refinement		
*R*[*F* ^2^ > 2σ(*F* ^2^)], *wR*(*F* ^2^), *S*	0.028, 0.058, 1.07	0.046, 0.102, 1.02
No. of reflections	2378	2821
No. of parameters	136	167
No. of restraints	3	6
H-atom treatment	H atoms treated by a mixture of independent and constrained refinement	H atoms treated by a mixture of independent and constrained refinement
Δρ_max_, Δρ_min_ (e Å^−3^)	0.29, −0.21	0.76, −0.64
Absolute structure	Flack *x* determined using 842 quotients [(*I* ^+^)−(*I* ^−^)]/[(*I* ^+^)+(*I* ^−^)] (Parsons *et al.*, 2013 ▸).	–
Absolute structure parameter	0.08 (5)	–

Computer programs: *CrysAlis PRO* (Agilent, 2012 ▸), *APEX2* and *SAINT* (Bruker, 2008 ▸), *SHELXS97* (Sheldrick, 2008 ▸), *SHELXL2014* (Sheldrick, 2015 ▸), *ORTEP-3 for Windows* (Farrugia, 2012 ▸), *QMol* (Gans & Shalloway, 2001 ▸), *DIAMOND* (Brandenburg, 2006 ▸) and *publCIF* (Westrip, 2010 ▸).

## supplementary crystallographic information

### (I) 5-Amino-N-phenyl-3H-1,2,4-dithiazol-3-iminium chloride Crystal data

**Table:**

C_8_H_8_N_3_S_2_^+^·Cl^−^	*D*_x_ = 1.578 Mg m^−^^3^
*M**_r_* = 245.74	Mo *K*α radiation, λ = 0.71073 Å
Orthorhombic, *P*2_1_2_1_2_1_	Cell parameters from 2635 reflections
*a* = 6.5702 (4) Å	θ = 2.3–27.3°
*b* = 10.8637 (7) Å	µ = 0.73 mm^−^^1^
*c* = 14.4964 (10) Å	*T* = 100 K
*V* = 1034.70 (12) Å^3^	Prism, yellow
*Z* = 4	0.15 × 0.02 × 0.02 mm
*F*(000) = 504	

### (I) 5-Amino-N-phenyl-3H-1,2,4-dithiazol-3-iminium chloride Data collection

**Table:**

Bruker SMART APEX CCD diffractometer	2378 independent reflections
Radiation source: fine-focus sealed tube	2185 reflections with *I* > 2σ(*I*)
Graphite monochromator	*R*_int_ = 0.044
φ and ω scans	θ_max_ = 27.5°, θ_min_ = 2.3°
Absorption correction: multi-scan (*SADABS*; Sheldrick, 1996)	*h* = −8→8
*T*_min_ = 0.898, *T*_max_ = 1.000	*k* = −14→14
9875 measured reflections	*l* = −18→18

### (I) 5-Amino-N-phenyl-3H-1,2,4-dithiazol-3-iminium chloride Refinement

**Table:**

Refinement on *F*^2^	Hydrogen site location: mixed
Least-squares matrix: full	H atoms treated by a mixture of independent and constrained refinement
*R*[*F*^2^ > 2σ(*F*^2^)] = 0.028	*w* = 1/[σ^2^(*F*_o_^2^) + (0.0242*P*)^2^ + 0.0389*P*] where *P* = (*F*_o_^2^ + 2*F*_c_^2^)/3
*wR*(*F*^2^) = 0.058	(Δ/σ)_max_ < 0.001
*S* = 1.07	Δρ_max_ = 0.29 e Å^−^^3^
2378 reflections	Δρ_min_ = −0.21 e Å^−^^3^
136 parameters	Absolute structure: Flack *x* determined using 842 quotients [(*I*^+^)-(*I*^-^)]/[(*I*^+^)+(*I*^-^)] (Parsons *et al.*, 2013).
3 restraints	Absolute structure parameter: 0.08 (5)

### (I) 5-Amino-N-phenyl-3H-1,2,4-dithiazol-3-iminium chloride Special details

**Table:**

Geometry. All esds (except the esd in the dihedral angle between two l.s. planes) are estimated using the full covariance matrix. The cell esds are taken into account individually in the estimation of esds in distances, angles and torsion angles; correlations between esds in cell parameters are only used when they are defined by crystal symmetry. An approximate (isotropic) treatment of cell esds is used for estimating esds involving l.s. planes.

### (I) 5-Amino-N-phenyl-3H-1,2,4-dithiazol-3-iminium chloride Fractional atomic coordinates and isotropic or equivalent isotropic displacement parameters (Å^2^)

**Table:**

	*x*	*y*	*z*	*U*_iso_*/*U*_eq_	
S1	1.07530 (11)	0.36767 (6)	0.26929 (5)	0.01784 (16)	
S2	0.85079 (11)	0.48700 (7)	0.22480 (5)	0.01919 (17)	
N1	1.0681 (4)	0.2920 (2)	0.44270 (15)	0.0173 (5)	
H1N	1.027 (4)	0.297 (3)	0.4995 (11)	0.021*	
H2N	1.182 (3)	0.255 (3)	0.4263 (19)	0.021*	
N2	0.8085 (3)	0.4255 (2)	0.40293 (14)	0.0140 (5)	
N3	0.5664 (4)	0.5602 (2)	0.33970 (16)	0.0175 (5)	
H3N	0.540 (4)	0.605 (2)	0.2910 (14)	0.021*	
C1	0.9739 (4)	0.3609 (2)	0.38220 (17)	0.0144 (6)	
C2	0.7320 (4)	0.4904 (3)	0.33453 (18)	0.0156 (6)	
C3	0.4216 (4)	0.5731 (2)	0.41161 (18)	0.0153 (6)	
C4	0.4307 (4)	0.5100 (2)	0.49499 (18)	0.0162 (6)	
H4	0.5415	0.4567	0.5083	0.019*	
C5	0.2751 (4)	0.5264 (3)	0.55831 (19)	0.0173 (6)	
H5	0.2780	0.4817	0.6146	0.021*	
C6	0.1161 (4)	0.6061 (3)	0.54155 (19)	0.0194 (6)	
H6	0.0121	0.6173	0.5863	0.023*	
C7	0.1093 (5)	0.6702 (3)	0.45801 (19)	0.0202 (7)	
H7	0.0006	0.7255	0.4458	0.024*	
C8	0.2609 (4)	0.6529 (3)	0.39322 (19)	0.0180 (6)	
H8	0.2554	0.6956	0.3361	0.022*	
Cl1	0.53467 (10)	0.70982 (6)	0.15949 (4)	0.01812 (16)	

### (I) 5-Amino-N-phenyl-3H-1,2,4-dithiazol-3-iminium chloride Atomic displacement parameters (Å^2^)

**Table:**

	*U*^11^	*U*^22^	*U*^33^	*U*^12^	*U*^13^	*U*^23^
S1	0.0186 (4)	0.0216 (3)	0.0133 (3)	0.0037 (3)	0.0025 (3)	0.0005 (3)
S2	0.0194 (4)	0.0250 (4)	0.0132 (3)	0.0046 (3)	0.0021 (3)	0.0022 (3)
N1	0.0167 (13)	0.0221 (12)	0.0131 (11)	0.0035 (12)	0.0024 (10)	0.0002 (10)
N2	0.0144 (13)	0.0146 (12)	0.0129 (11)	−0.0016 (10)	−0.0004 (9)	−0.0006 (9)
N3	0.0181 (12)	0.0194 (12)	0.0151 (11)	0.0040 (11)	0.0019 (11)	0.0045 (10)
C1	0.0150 (14)	0.0150 (13)	0.0133 (12)	−0.0036 (12)	0.0014 (11)	−0.0008 (11)
C2	0.0167 (14)	0.0161 (13)	0.0138 (12)	−0.0033 (12)	0.0005 (12)	−0.0019 (12)
C3	0.0134 (14)	0.0152 (13)	0.0172 (13)	−0.0023 (12)	0.0017 (12)	−0.0031 (11)
C4	0.0155 (14)	0.0155 (13)	0.0175 (13)	−0.0007 (13)	−0.0012 (12)	−0.0005 (10)
C5	0.0184 (15)	0.0195 (14)	0.0138 (13)	−0.0044 (12)	−0.0015 (11)	−0.0005 (11)
C6	0.0152 (16)	0.0220 (15)	0.0210 (15)	−0.0017 (12)	0.0044 (12)	−0.0035 (12)
C7	0.0166 (18)	0.0188 (14)	0.0252 (16)	0.0036 (12)	−0.0012 (12)	−0.0039 (12)
C8	0.0203 (16)	0.0162 (14)	0.0176 (14)	−0.0004 (12)	−0.0022 (12)	0.0003 (11)
Cl1	0.0179 (4)	0.0221 (3)	0.0144 (3)	−0.0015 (3)	−0.0009 (3)	0.0030 (3)

### (I) 5-Amino-N-phenyl-3H-1,2,4-dithiazol-3-iminium chloride Geometric parameters (Å, º)

**Table:**

S1—C1	1.769 (3)	C3—C4	1.391 (4)
S1—S2	2.0669 (10)	C3—C8	1.392 (4)
S2—C2	1.772 (3)	C4—C5	1.386 (4)
N1—C1	1.309 (3)	C4—H4	0.9500
N1—H1N	0.869 (13)	C5—C6	1.378 (4)
N1—H2N	0.881 (13)	C5—H5	0.9500
N2—C2	1.317 (3)	C6—C7	1.398 (4)
N2—C1	1.328 (3)	C6—H6	0.9500
N3—C2	1.328 (3)	C7—C8	1.382 (4)
N3—C3	1.418 (3)	C7—H7	0.9500
N3—H3N	0.875 (12)	C8—H8	0.9500
			
C1—S1—S2	92.63 (9)	C8—C3—N3	115.5 (2)
C2—S2—S1	92.72 (10)	C5—C4—C3	118.7 (3)
C1—N1—H1N	117.0 (19)	C5—C4—H4	120.6
C1—N1—H2N	119 (2)	C3—C4—H4	120.6
H1N—N1—H2N	123 (3)	C6—C5—C4	121.6 (3)
C2—N2—C1	115.1 (2)	C6—C5—H5	119.2
C2—N3—C3	130.4 (2)	C4—C5—H5	119.2
C2—N3—H3N	116 (2)	C5—C6—C7	119.3 (3)
C3—N3—H3N	114 (2)	C5—C6—H6	120.3
N1—C1—N2	122.5 (2)	C7—C6—H6	120.3
N1—C1—S1	117.8 (2)	C8—C7—C6	119.9 (3)
N2—C1—S1	119.7 (2)	C8—C7—H7	120.1
N2—C2—N3	125.2 (2)	C6—C7—H7	120.1
N2—C2—S2	119.8 (2)	C7—C8—C3	120.1 (3)
N3—C2—S2	115.1 (2)	C7—C8—H8	120.0
C4—C3—C8	120.4 (3)	C3—C8—H8	120.0
C4—C3—N3	124.1 (2)		
			
C2—N2—C1—N1	179.8 (3)	C2—N3—C3—C4	0.2 (4)
C2—N2—C1—S1	0.4 (3)	C2—N3—C3—C8	−178.3 (3)
S2—S1—C1—N1	−179.9 (2)	C8—C3—C4—C5	1.2 (4)
S2—S1—C1—N2	−0.5 (2)	N3—C3—C4—C5	−177.2 (2)
C1—N2—C2—N3	179.2 (2)	C3—C4—C5—C6	−1.9 (4)
C1—N2—C2—S2	−0.1 (3)	C4—C5—C6—C7	1.2 (4)
C3—N3—C2—N2	−7.6 (5)	C5—C6—C7—C8	0.2 (4)
C3—N3—C2—S2	171.6 (2)	C6—C7—C8—C3	−0.9 (4)
S1—S2—C2—N2	−0.2 (2)	C4—C3—C8—C7	0.2 (4)
S1—S2—C2—N3	−179.5 (2)	N3—C3—C8—C7	178.7 (2)

### (I) 5-Amino-N-phenyl-3H-1,2,4-dithiazol-3-iminium chloride Hydrogen-bond geometry (Å, º)

**Table:**

*D*—H···*A*	*D*—H	H···*A*	*D*···*A*	*D*—H···*A*
N1—H1*N*···Cl1^i^	0.87 (2)	2.36 (2)	3.215 (2)	170 (3)
N1—H2*N*···Cl1^ii^	0.88 (2)	2.29 (2)	3.131 (3)	159 (3)
N3—H3*N*···Cl1	0.88 (2)	2.22 (2)	3.084 (2)	169 (2)

Symmetry codes: (i) −*x*+3/2, −*y*+1, *z*+1/2; (ii) −*x*+2, *y*−1/2, −*z*+1/2.

### (II) 5-Amino-N-(4-chlorophenyl)-3H-1,2,4-dithiazol-3-iminium chloride monohydrate Crystal data

**Table:**

C_8_H_7_ClN_3_S_2_^+^·Cl^−^·H_2_O	*F*(000) = 1216
*M**_r_* = 298.20	*D*_x_ = 1.618 Mg m^−^^3^
Monoclinic, *C*2/*c*	Mo *K*α radiation, λ = 0.71073 Å
*a* = 17.0581 (7) Å	Cell parameters from 4628 reflections
*b* = 14.1660 (7) Å	θ = 2.4–27.5°
*c* = 10.3215 (4) Å	µ = 0.85 mm^−^^1^
β = 101.084 (4)°	*T* = 100 K
*V* = 2447.61 (19) Å^3^	Prism, yellow
*Z* = 8	0.20 × 0.10 × 0.05 mm

### (II) 5-Amino-N-(4-chlorophenyl)-3H-1,2,4-dithiazol-3-iminium chloride monohydrate Data collection

**Table:**

Agilent SuperNova Dual diffractometer with an Atlas detector	2821 independent reflections
Radiation source: SuperNova (Mo) X-ray Source	2142 reflections with *I* > 2σ(*I*)
Mirror monochromator	*R*_int_ = 0.064
Detector resolution: 10.4041 pixels mm^-1^	θ_max_ = 27.5°, θ_min_ = 2.4°
ω scan	*h* = −22→22
Absorption correction: multi-scan (*CrysAlis PRO*; Agilent, 2012)	*k* = −18→18
*T*_min_ = 0.748, *T*_max_ = 1.000	*l* = −13→13
19709 measured reflections	

### (II) 5-Amino-N-(4-chlorophenyl)-3H-1,2,4-dithiazol-3-iminium chloride monohydrate Refinement

**Table:**

Refinement on *F*^2^	6 restraints
Least-squares matrix: full	Hydrogen site location: mixed
*R*[*F*^2^ > 2σ(*F*^2^)] = 0.046	H atoms treated by a mixture of independent and constrained refinement
*wR*(*F*^2^) = 0.102	*w* = 1/[σ^2^(*F*_o_^2^) + (0.0237*P*)^2^ + 10.8824*P*] where *P* = (*F*_o_^2^ + 2*F*_c_^2^)/3
*S* = 1.02	(Δ/σ)_max_ = 0.001
2821 reflections	Δρ_max_ = 0.76 e Å^−^^3^
167 parameters	Δρ_min_ = −0.64 e Å^−^^3^

### (II) 5-Amino-N-(4-chlorophenyl)-3H-1,2,4-dithiazol-3-iminium chloride monohydrate Special details

**Table:**

Geometry. All esds (except the esd in the dihedral angle between two l.s. planes) are estimated using the full covariance matrix. The cell esds are taken into account individually in the estimation of esds in distances, angles and torsion angles; correlations between esds in cell parameters are only used when they are defined by crystal symmetry. An approximate (isotropic) treatment of cell esds is used for estimating esds involving l.s. planes.

### (II) 5-Amino-N-(4-chlorophenyl)-3H-1,2,4-dithiazol-3-iminium chloride monohydrate Fractional atomic coordinates and isotropic or equivalent isotropic displacement parameters (Å^2^)

**Table:**

	*x*	*y*	*z*	*U*_iso_*/*U*_eq_	Occ. (<1)
Cl1	0.55093 (5)	0.90087 (7)	−0.09408 (7)	0.0413 (2)	
Cl2	0.79427 (5)	0.93836 (6)	1.03317 (7)	0.0315 (2)	0.9327 (18)
Cl2'	0.8457 (8)	0.9762 (9)	1.0388 (10)	0.0315 (2)	0.0673 (18)
S1	0.70602 (5)	0.75085 (6)	0.81299 (7)	0.02725 (19)	
S2	0.65448 (5)	0.65361 (6)	0.67312 (7)	0.0304 (2)	
N1	0.73581 (17)	0.9221 (2)	0.7316 (2)	0.0307 (6)	
H1N	0.740 (2)	0.9640 (19)	0.671 (3)	0.037*	
H2N	0.7550 (19)	0.932 (3)	0.8156 (13)	0.037*	
N2	0.67993 (14)	0.81976 (17)	0.5669 (2)	0.0216 (5)	
N3	0.61963 (15)	0.70381 (19)	0.4213 (2)	0.0253 (6)	
H3N	0.5993 (18)	0.6468 (11)	0.415 (3)	0.030*	
C1	0.70728 (17)	0.8388 (2)	0.6944 (3)	0.0234 (6)	
C2	0.65147 (16)	0.7342 (2)	0.5421 (3)	0.0222 (6)	
C3	0.60695 (16)	0.7557 (2)	0.3010 (3)	0.0208 (6)	
C4	0.60976 (17)	0.8531 (2)	0.2941 (3)	0.0246 (6)	
H4	0.6230	0.8895	0.3726	0.029*	
C5	0.59310 (17)	0.8977 (2)	0.1718 (3)	0.0258 (6)	
H5	0.5950	0.9645	0.1662	0.031*	
C6	0.57366 (16)	0.8435 (2)	0.0582 (3)	0.0252 (7)	
C7	0.57171 (17)	0.7464 (2)	0.0638 (3)	0.0277 (7)	
H7	0.5593	0.7103	−0.0150	0.033*	
C8	0.58805 (17)	0.7020 (2)	0.1854 (3)	0.0258 (6)	
H8	0.5864	0.6351	0.1904	0.031*	
O1W	0.54036 (18)	0.5195 (2)	0.3920 (3)	0.0455 (7)	0.9327 (18)
H1O	0.5860 (11)	0.495 (3)	0.419 (4)	0.068*	
H2O	0.5075 (16)	0.493 (3)	0.430 (4)	0.068*	
O1W'	0.503 (2)	0.485 (3)	0.336 (4)	0.0455 (7)	0.0673 (18)

### (II) 5-Amino-N-(4-chlorophenyl)-3H-1,2,4-dithiazol-3-iminium chloride monohydrate Atomic displacement parameters (Å^2^)

**Table:**

	*U*^11^	*U*^22^	*U*^33^	*U*^12^	*U*^13^	*U*^23^
Cl1	0.0453 (5)	0.0588 (6)	0.0198 (4)	0.0229 (4)	0.0062 (3)	0.0110 (4)
Cl2	0.0394 (5)	0.0378 (5)	0.0172 (4)	0.0038 (4)	0.0051 (3)	0.0024 (3)
Cl2'	0.0394 (5)	0.0378 (5)	0.0172 (4)	0.0038 (4)	0.0051 (3)	0.0024 (3)
S1	0.0329 (4)	0.0323 (4)	0.0169 (4)	0.0045 (3)	0.0056 (3)	0.0028 (3)
S2	0.0432 (5)	0.0290 (4)	0.0193 (4)	−0.0042 (4)	0.0068 (3)	0.0052 (3)
N1	0.0455 (17)	0.0270 (15)	0.0170 (12)	−0.0015 (13)	−0.0003 (12)	−0.0009 (11)
N2	0.0220 (12)	0.0239 (13)	0.0183 (12)	0.0001 (10)	0.0027 (9)	0.0019 (10)
N3	0.0288 (14)	0.0279 (14)	0.0191 (12)	−0.0079 (11)	0.0040 (10)	0.0012 (10)
C1	0.0233 (15)	0.0290 (16)	0.0183 (13)	0.0042 (13)	0.0052 (11)	0.0025 (12)
C2	0.0201 (14)	0.0293 (17)	0.0184 (14)	0.0012 (12)	0.0070 (11)	0.0037 (12)
C3	0.0175 (13)	0.0280 (16)	0.0174 (13)	−0.0047 (12)	0.0045 (10)	0.0013 (11)
C4	0.0228 (15)	0.0331 (17)	0.0173 (14)	0.0008 (13)	0.0025 (11)	−0.0013 (12)
C5	0.0221 (15)	0.0301 (17)	0.0246 (15)	0.0049 (13)	0.0032 (12)	0.0049 (13)
C6	0.0167 (14)	0.0415 (19)	0.0171 (13)	0.0066 (13)	0.0025 (11)	0.0053 (13)
C7	0.0215 (15)	0.0423 (19)	0.0191 (14)	−0.0025 (14)	0.0034 (11)	−0.0055 (13)
C8	0.0241 (15)	0.0293 (17)	0.0240 (15)	−0.0074 (13)	0.0044 (12)	−0.0012 (12)
O1W	0.0394 (17)	0.0353 (17)	0.066 (2)	0.0025 (13)	0.0211 (15)	0.0151 (14)
O1W'	0.0394 (17)	0.0353 (17)	0.066 (2)	0.0025 (13)	0.0211 (15)	0.0151 (14)

### (II) 5-Amino-N-(4-chlorophenyl)-3H-1,2,4-dithiazol-3-iminium chloride monohydrate Geometric parameters (Å, º)

**Table:**

Cl1—C6	1.746 (3)	C3—C8	1.399 (4)
S1—C1	1.749 (3)	C4—C5	1.390 (4)
S1—S2	2.0657 (11)	C4—H4	0.9500
S2—C2	1.763 (3)	C5—C6	1.387 (4)
N1—C1	1.306 (4)	C5—H5	0.9500
N1—H1N	0.876 (10)	C6—C7	1.377 (5)
N1—H2N	0.876 (10)	C7—C8	1.384 (4)
N2—C2	1.312 (4)	C7—H7	0.9500
N2—C1	1.337 (4)	C8—H8	0.9500
N3—C2	1.332 (4)	O1W—H1O	0.850 (10)
N3—C3	1.423 (4)	O1W—H2O	0.835 (10)
N3—H3N	0.876 (10)	O1W'—O1W'^i^	1.75 (9)
C3—C4	1.384 (4)		
			
C1—S1—S2	92.68 (11)	C8—C3—N3	115.8 (3)
C2—S2—S1	92.84 (11)	C3—C4—C5	119.8 (3)
C1—N1—H1N	119 (2)	C3—C4—H4	120.1
C1—N1—H2N	119 (2)	C5—C4—H4	120.1
H1N—N1—H2N	122 (3)	C6—C5—C4	119.4 (3)
C2—N2—C1	115.1 (2)	C6—C5—H5	120.3
C2—N3—C3	128.0 (3)	C4—C5—H5	120.3
C2—N3—H3N	117 (2)	C7—C6—C5	121.4 (3)
C3—N3—H3N	115 (2)	C7—C6—Cl1	120.0 (2)
N1—C1—N2	120.7 (3)	C5—C6—Cl1	118.7 (3)
N1—C1—S1	119.5 (2)	C6—C7—C8	119.4 (3)
N2—C1—S1	119.8 (2)	C6—C7—H7	120.3
N2—C2—N3	123.4 (3)	C8—C7—H7	120.3
N2—C2—S2	119.6 (2)	C7—C8—C3	119.9 (3)
N3—C2—S2	117.0 (2)	C7—C8—H8	120.0
C4—C3—C8	120.2 (3)	C3—C8—H8	120.0
C4—C3—N3	124.0 (3)	H1O—O1W—H2O	108.5 (17)
			
C2—N2—C1—N1	−178.8 (3)	C2—N3—C3—C8	167.1 (3)
C2—N2—C1—S1	1.9 (4)	C8—C3—C4—C5	0.5 (4)
S2—S1—C1—N1	179.7 (2)	N3—C3—C4—C5	−177.2 (3)
S2—S1—C1—N2	−1.0 (2)	C3—C4—C5—C6	0.1 (4)
C1—N2—C2—N3	178.0 (3)	C4—C5—C6—C7	−1.0 (4)
C1—N2—C2—S2	−1.9 (3)	C4—C5—C6—Cl1	178.7 (2)
C3—N3—C2—N2	−3.0 (5)	C5—C6—C7—C8	1.2 (4)
C3—N3—C2—S2	176.9 (2)	Cl1—C6—C7—C8	−178.5 (2)
S1—S2—C2—N2	1.1 (2)	C6—C7—C8—C3	−0.5 (4)
S1—S2—C2—N3	−178.9 (2)	C4—C3—C8—C7	−0.3 (4)
C2—N3—C3—C4	−15.1 (5)	N3—C3—C8—C7	177.6 (3)

Symmetry code: (i) −*x*+1, *y*, −*z*+1/2.

### (II) 5-Amino-N-(4-chlorophenyl)-3H-1,2,4-dithiazol-3-iminium chloride monohydrate Hydrogen-bond geometry (Å, º)

**Table:**

*D*—H···*A*	*D*—H	H···*A*	*D*···*A*	*D*—H···*A*
N1—H1*N*···Cl2^ii^	0.88 (3)	2.30 (3)	3.144 (3)	161 (3)
N1—H2*N*···Cl2	0.88 (2)	2.22 (1)	3.089 (2)	172 (4)
N3—H3*N*···O1*W*	0.88 (2)	2.06 (2)	2.927 (4)	174 (3)
O1*W*—H2*O*···O1*W*^iii^	0.84 (3)	2.29 (4)	2.884 (4)	128 (3)
O1*W*—H1*O*···Cl2^iv^	0.85 (3)	2.16 (3)	3.005 (3)	170 (3)

Symmetry codes: (ii) *x*, −*y*+2, *z*−1/2; (iii) −*x*+1, −*y*+1, −*z*+1; (iv) −*x*+3/2, *y*−1/2, −*z*+3/2.
